# Comparative efficacy of linezolid and vancomycin for endotracheal tube MRSA biofilms from ICU patients

**DOI:** 10.1186/s13054-019-2523-5

**Published:** 2019-07-10

**Authors:** Laia Fernández-Barat, Ana Motos, Mauro Panigada, Francisco Álvarez-Lerma, Lucía Viña, Ruben Lopez-Aladid, Adrian Ceccato, Gianluigi Li Bassi, David P. Nicolau, Yuli Lopez, Laura Muñoz, Laura Guerrero, Dolors Soy, Trinidad Israel, Pedro Castro, Antoni Torres

**Affiliations:** 1 Cellex Laboratory, CibeRes ((Centro de Investigación Biomédica en Red de Enfermedades Respiratorias, 06/06/0028), Institut d’Investigacions Biomèdiques August Pi i Sunyer (IDIBAPS), School of Medicine, University of Barcelona, C/ Casanova 143, 08036, Cellex laboratory, Barcelona, Spain; 2 Respiratory Intensive Care Unit Pneumology Department, Hospital Clínic, Barcelona, Spain; 3 Department of Anesthesiology, Intensive Care and Emergency, Fondazione IRCCS Ca’ Granda, Ospedale Maggiore Policlinico, Milan, Italy; 4 Critical Care Department, Hospital del Mar, Critical Illness Research Group (GREPAC), Hospital del Mar Medical Research Institute (IMIM), Barcelona, Spain; 5 Servicio de Medicina Intensiva, Hospital Universitario Central de Asturias, Oviedo, Spain; 6 Center for Anti-Infective Research and Development, Hartford Hospital, Hartford, CT USA; 7 Microbiology Department, Hospital Clínic, CRESIB ISglobal, Barcelona, Spain; 8 Medical Intensive Care Unit, Hospital Clínic, Barcelona, Spain; 9 Pharmacy Service, Division of Medicines, Hospital Clínic, Barcelona, Spain

**Keywords:** Endotracheal tube, Biofilm, Methicillin-resistant *Staphylococcus aureus*, Linezolid, Vancomycin, Respiratory infection

## Abstract

**Purpose:**

To compare the efficacy of systemic treatment with linezolid (LNZ) versus vancomycin (VAN) on methicillin-resistant *Staphylococcus aureus* (MRSA) burden and eradication in endotracheal tube (ETT) biofilm and ETT cuff from orotracheally intubated patients with MRSA respiratory infection.

**Methods:**

Prospective observational clinical study was carried out at four European tertiary hospitals. Plasma and endotracheal aspirate (ETA) levels of LNZ and VAN were determined 72 h after treatment initiation through high-performance liquid chromatography or bioassay. LNZ or VAN concentration in the ETT biofilm and MRSA burden and eradication was determined upon extubation. The minimum inhibitory concentration (MIC) for LNZ and VAN was assessed by E-test strips (Biomerieux®). Scanning electron microscopy images were obtained, and ETT biofilm thickness was compared between groups.

**Results:**

Twenty-five patients, 15 treated with LNZ and 10 with VAN, were included in the study. LNZ presented a significantly higher concentration (μg/mL) than VAN in ETT biofilm (72.8 [1.3–127.1] vs 0.4 [0.4–1.3], *p* < 0.001), although both drugs achieved therapeutic plasma levels 72 h after treatment initiation. Systemic treatment with LNZ achieved lower ETT cuff MRSA burdens than systemic treatment with VAN. Indeed, LNZ increased the MRSA eradication rate in ETT cuff compared with VAN (LNZ 75%, VAN 20%, *p* = 0.031).

**Conclusions:**

In ICU patients with MRSA respiratory infection intubated for long periods, systemic treatment with LNZ obtains a greater beneficial effect than VAN in limiting MRSA burden in ETT cuff.

**Electronic supplementary material:**

The online version of this article (10.1186/s13054-019-2523-5) contains supplementary material, which is available to authorized users.

## Introduction

In orotracheally intubated critically ill patients, bacteria from the oropharyngeal or gastric microbiota can rapidly colonize the lower respiratory airways passing over the endotracheal tube cuff (ETT cuff) and colonizing the inner ETT surface by forming biofilms [1, 2]. Thus, these patients are especially vulnerable to developing a respiratory infection caused by a nosocomial pathogen such as *Staphylococcus aureus*, in either its methicillin-sensitive (MSSA) or methicillin-resistant (MRSA) form.

*S. aureus* has recently been identified as the second most frequently isolated microorganism responsible for intensive care unit (ICU)-acquired pneumonia, of which 29% of cases are MRSA [3]. The current clinical guidelines for hospital-acquired pneumonia (HAP) and ventilator-associated pneumonia (VAP) published by the Infectious Diseases Society of America and the American Thoracic Society (IDSA/ATS) recommend either intravenous vancomycin (VAN) or linezolid (LNZ) as first-line treatment for MRSA ICU-acquired respiratory infection [4], while the International ERS/ESICM/ESCMID/ALAT guidelines prefer LNZ than VAN [5]. However, the microbiological confirmation of MRSA cultures takes at least 48–72 h, a sufficient time-lapse for ETT biofilm formation.

ETT biofilm formation is currently considered one of the multiple factors that can lead to VAP or its relapse [2]. While several preventive strategies have targeted ETT biofilm eradication [6–9], none of them has achieved 100% success. The silver-coated ETT and mucus shaver have demonstrated improvements in reducing ETT biofilm in randomized clinical trials but still present certain limitations which may delay their implementation in clinical practice [7, 8]. Although biofilms exhibit antimicrobial tolerance [10], little is known about how antimicrobials affect biofilm formation during endotracheal intubation. In a previous study in pigs with MRSA pneumonia, we found that those treated with LNZ achieved better pharmacokinetic and pharmacodynamic indices in serum and lung tissue [11], very high levels of LNZ within ETT biofilm, and a lower ETT biofilm MRSA burden in comparison with untreated controls; however, similar rates were not found in the VAN group [12, 13]. What is more, in a study in piglets, Luna et al. found that LNZ was associated with a lower pathology score, better survival, and a trend towards better clearance of MRSA in comparison with glycopeptides [14]. Since it is well known that findings in pigs are not always reproducible in humans [15], we designed a clinical observational study in ICU patients to assess this issue.

Our study aimed to determine the effect of systemic treatment with LNZ vs VAN on ETT biofilm from ICU patients with respiratory MRSA infection, including LNZ and VAN concentration measurements within plasma and endotracheal aspirate (ETA) 72 h after treatment initiation and within ETT biofilm upon extubation.

## Materials and methods

### Patients

The study was conducted at the medical and surgical ICUs of four university hospitals in southern Europe, three in Spain and one in Italy. The following hospitals enrolled patients: Hospital Clinic, Barcelona, Spain (including the following ICUs: Respiratory, Medical, Surgical, Cardiovascular and Hepatic), Hospital del Mar (Critical Care Dept), also in Barcelona, Spain, Hospital Universitario Central de Asturias, in Oviedo, Spain (Intensive Medicine Service), and the Fondazione IRCCS Ca’ Granda, Ospedale Maggiore Policlinico, in Milan, Italy (Adult Intensive Care).

Data were prospectively collected from September 2013 to December 2016. The investigators made daily rounds in all ICUs. Patients were included consecutively, and only the first episode was analyzed. All patients were over 18 and had respiratory infection due to *S. aureus* (confirmed microbiologically) with ≥ 48 h of orotracheal intubation and ≥ 48 h of treatment with either LNZ or VAN. Patients with severe immunosuppression (neutropenia after chemotherapy or hematopoietic stem cell transplantation, drug-induced immunosuppression in solid–organ transplant or cytotoxic therapy, and HIV infection-related disorders) were not registered.

The study was carried out in compliance with the Declaration of Helsinki (current version, Fortaleza, Brazil, October 2013) and was conducted in accordance with the requirements of the 2007 Spanish Biomedical Research Act. The study was approved by the institution’s Internal Review Board (registry number 2012/7927). Written informed consent was obtained from patients or their next of kin.

### Definitions

The clinical suspicion of pneumonia was based on clinical criteria. We considered VAP in patients with previous invasive mechanical ventilation for 48 h or more. Patients were classified as VAP or non-ventilator ICU-acquired pneumonia (i.e., cases that do not meet the VAP criteria) [16]. Early-onset VAP was defined as occurring within the first 4 days of invasive mechanical ventilation. The respiratory infection was considered ventilator-associated tracheobronchitis when at least two of the aforementioned criteria for pneumonia were found in the absence of radiographic signs of new pneumonia [17]. Severe community-acquired pneumonia (SCAP) was defined according to the 2007 IDSA/ATS guidelines [18] and as previously defined [19]. All SCAP patients included required invasive mechanical ventilation.

### Microbiology and antimicrobial treatment

The microbiological evaluation has been extensively addressed in previous reports [20]. Microbial identification and susceptibility testing were performed by standard methods [21, 22].

The initial empiric antimicrobial treatment was administered according to local adaptations of international guidelines [5] and subsequently revised according to the microbiology results.

### Data collection and severity assessment

All relevant data were collected at admission and at the onset of pneumonia from the medical records and bedside flow charts, including clinical, laboratory, radiological, and microbiological information. Patients were followed until the end of mechanical ventilation.

The severity assessment included the APACHE-II [23] and the Sequential Organ Failure Assessment (SOFA) [24] score on ICU admission at microbial diagnosis and at orotracheal extubation.

### Endotracheal tube preparation and microbiology analysis

All ETTs from the patients included were collected and stored at − 80 °C until analysis. All ETTs were number coded so that the investigators would be blind to treatment group allocation during the analysis. For the first time, we also included the microbiological culture of the ETT cuff. The ETT cuff was dissected and microbiologically processed before rinsing the outer surface and slicing the ETT, following our methodology published elsewhere [12]. Both ETT cuff and ETT were sonicated before microbiological cultures. Bacterial growth was quantified and reported as logarithmic scale of colony-forming units per milliliter (log_10_ CFU/mL). Susceptibility to oxacillin, linezolid, and vancomycin was assessed for all *S. aureus* strains isolated from ETT through E-test strips (Biomerieux, France) using ATCC25923 strain as standard laboratory testing control strain, following the manufacturer’s recommendations.

### Determination of MLST from Sanger data and phylogenetic analyses

The Sanger sequences were used to obtain the allelic profile of seven *S. aureus* housekeeping genes (arcC, aroE, glpF, gmk, pta, tpi, yqiL). The genes were concatenated by the MLST.net database. The MLST results were compared to references in NCBI and the *S. aureus* MLST database in order to assign sequence type (ST). The MLST results were compared against the MLST database (https://pubmlst.org/saureus/) using comparative eBURST V3 software employing the BURST algorithm [25]. Accessory gene regulator (agr) type I, II, III, IV, or V was confirmed by conventional polymerase chain reaction (PCR) using previously described primers and reaction conditions [26].

### Antibiotic concentration in biological matrixes

LNZ or VAN concentrations in biological matrixes (i.e., plasma, ETA, and ETT biofilm) were determined using high-performance liquid chromatography (HPLC) as previously described [11]. To release antibiotics from ETA and biofilm and to perform the HPLC, we applied our methodology previously described elsewhere [12]. The lower limit of detection of HPLC was 2.5 μg mL^− 1^ for both antibiotics. When the sample was below detection limit (BDL), the value assigned was 1.25 μg/mL.

A bioassay was alternatively performed for the detection of vancomycin, as previously reported [12]. *Bacillus subtillis* (ATCC 6633) in Mueller-Hinton Agar was used for the analysis. The lower limit of detection of the vancomycin bioassay was 0.70 μg mL^− 1^. When the sample was BDL, the value assigned was 0.35 μg/mL.

### Scanning electron microscopy

Biofilm was imaged and thickness measured via scanning electron microscopy (SEM) [12]. Briefly, a 1-cm-long hemisection of the ETT distal dependent parts were fixed, dehydrated in graded alcohol series, dried using a polaron critical point drying apparatus, and mounted on commercial SEM stubs (Ted Pella, Inc. Spain). To avoid charge artifacts, the section was sputter-coated with a gold thin layer (sc 510, Fisons Instrument, East Sussex, UK) and carefully silver painted. Samples were imaged via a scanning electron microscope (JEOL JSM 7001F FEG, Japan), and micrographs were recorded on a personal computer. We measured minimal, maximal, and mean biofilm thickness using dedicated software (ImageJ, Wayne Rasband, NIH, USA).

### Statistical analysis

Categorical variables were reported as number (%), while continuous variables were reported as mean SD or median (interquartile range, IQR), if the distribution was normal or non-normal respectively. Continuous variables between groups were compared using the one-way analysis of variance (ANOVA) or the Kruskal-Wallis test as appropriate. Post hoc pairwise comparisons were carried out via Tukey’s honestly significant difference (HSD) test. Paired samples were compared with the paired *t*-test or non-parametric Wilcoxon signed-rank test when appropriate. Spearman’s correlation analyses were performed to determine associations between continuous variables. A two-sided *p* value ≤ 0.05 was considered statistically significant. Data were processed with IBM SPSS Statistic for Windows, version 22.0 (IBM Corporation, Armonk, NY, USA).

## Results

### Subjects

**Fig. 1:**
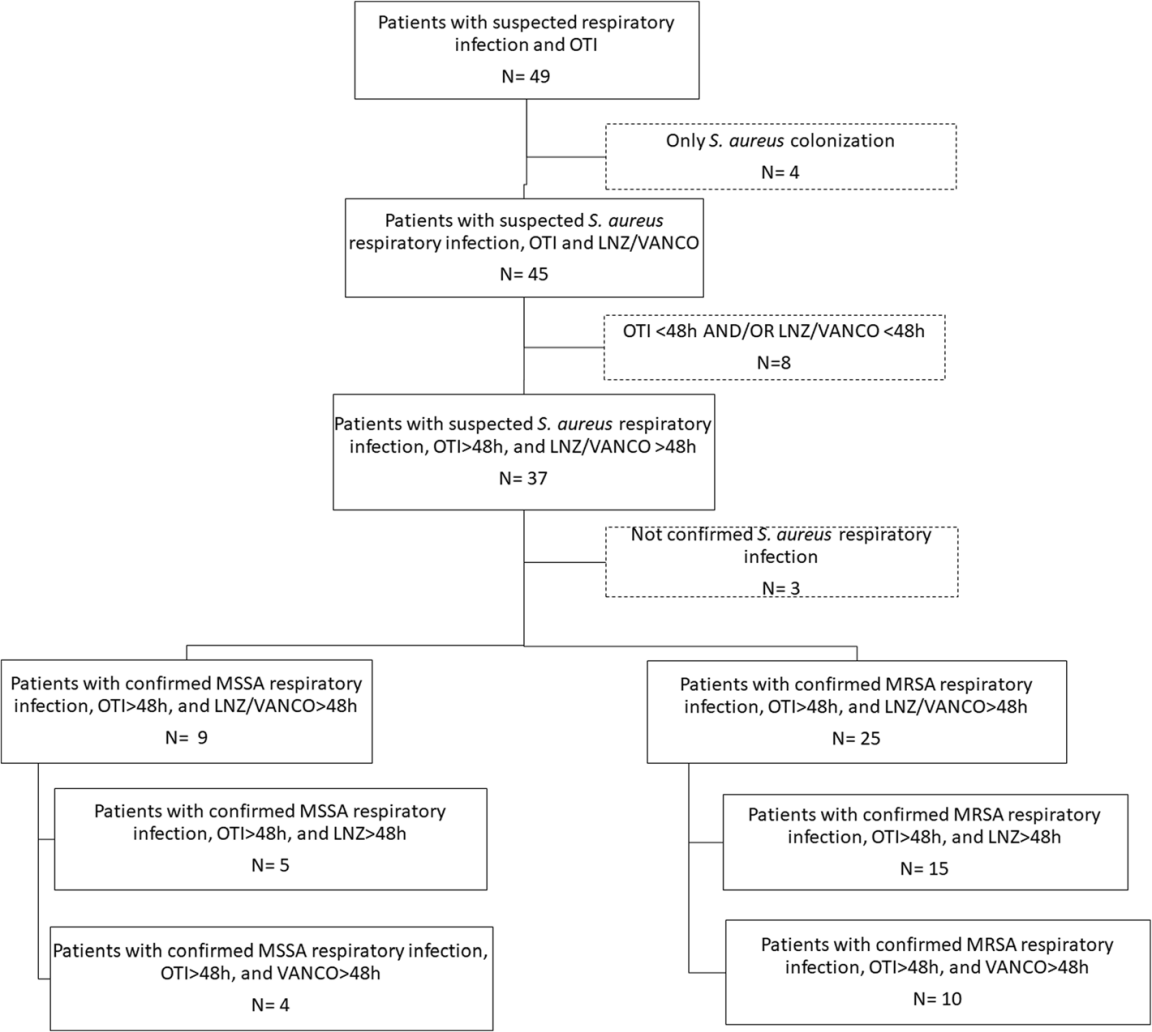
Flow chart of screened, excluded, and included patients. OTI, orotracheal intubation

From October 2013 to December 2016, 34 orotracheally intubated patients with microbiologically confirmed *S. aureus* respiratory infection were consecutively screened for this study, with 20 receiving LNZ and 14 VAN for more than 48 h. Twenty-five of them (15 in the LNZ group and 10 in the VAN group) had MRSA respiratory infection and were included in the analysis (Fig. 1). No significant differences were found between groups in terms of baseline clinical characteristics on ICU admission (Table 1). Nor were any differences found at microbial diagnosis with regard to type of respiratory infection (predominantly ventilator-associated pneumonia, VAP), type of sample (predominantly BAS), MRSA load, radiological diagnosis (predominantly bilateral pneumonia), or severity according to APACHE II and SOFA scores (Table 2).

**Table 1:** Baseline clinical characteristics of the population

MRSA respiratory ICU infection (*n* = 25)	Linezolid (*n* = 15)	Vancomycin (*n* = 10)	*p* value
Age (years)	63.4[57.0–69.7]	56.1[32.8–66.6]	0.13
Male sex	12 (80.0)	7 (70.0)	0.65
APACHE II ICU admission	21.0[15.5–23.5]	18.0[13.3–22.0]	0.24
SOFA ICU admission	7.0[3.0–9.5]	7.5[4.8–12.3]	0.29
Coexisting illness/comorbidities			
CLD	8 (53.3)	2 (22.2)	0.21
COPD	4	1	
Bronchiectasis	1	0	
Asthma	1	1	
Lung cancer	3	0	
Diabetes	4 (25.0)	0	0.13
Substance use behavior			
Alcohol use disorder	5 (33.3)	0	0.06
Current smoker	8 (53.3)	2 (20.0)	0.21
Previous systemic antibiotics	10 (66.7)	6 (60.0)	1.0
Previous colonization	6 (40.0)	6 (60.0)	0.43
Previous corticosteroids (inhaled)	2 (13.3)	1 (10.0)	1.0

Data are presented as median and interquartile range [percentiles 25th–75th] or *n* (%). *APACHE II* Acute Physiology and Chronic Health Evaluation, *ICU* intensive care unit, *SOFA* Sequential Organ Failure Assessment, *CLD* chronic lung disease, *COPD* chronic obstructive pulmonary disease. One patient had both COPD and lung cancer

**Table 2:** Characteristics of patients at microbial diagnosis

MRSA respiratory ICU infection (*n* = 25)	Linezolid (15)	Vancomycin (10)	*p* value
Respiratory infection			0.76
Tracheobronchitis	2	2	
HAP	2	2	
VAP	8	5	
Early onset VAP	4	4	
Late onset VAP	4	1	
Severe CAP	3	1	
Type of sample			0.24
BAL	2	0	
BAS	10	10	
ETA	2	0	
Pleural fluid	1	0	
Microbial diagnostic			
Log CFU/mL	6.0[4.0–6.0]	5.0[4.0–6.0]	0.40
Polymicrobial respiratory infection	7	3	0.69
Radiographic consolidation			0.34
None	2	0	
Monolateral	2	3	
Bilateral	11	7	
Severity scores			
APACHE II ICU at microbial diagnosis	17.0[11.5–22.8]	17.5[6.5–22.3]	0.58
SOFA ICU at microbial diagnosis	8.0[1.8–10.0]	7.0[5.5–10.8]	0.55

Data are presented as median and interquartile range [percentiles 25th–75th] or *n* (%). *ICU* intensive care unit, *HAP* hospital-acquired pneumonia, *VAP* ventilator-associated pneumonia, *CAP* community-acquired pneumonia, *BAL* bronchoalveolar lavage, *BAS* tracheobronchial aspirates, *ETA* endotracheal aspirates, *APACHE II* Acute Physiology and Chronic Health Evaluation, *SOFA* Sequential Organ Failure Assessment, *MRSA* methicillin-resistant *S. aureus*, *CFU* colony-forming units

### Quantitative microbiology assessment of ETT and ETT cuff

**Fig. 2:**
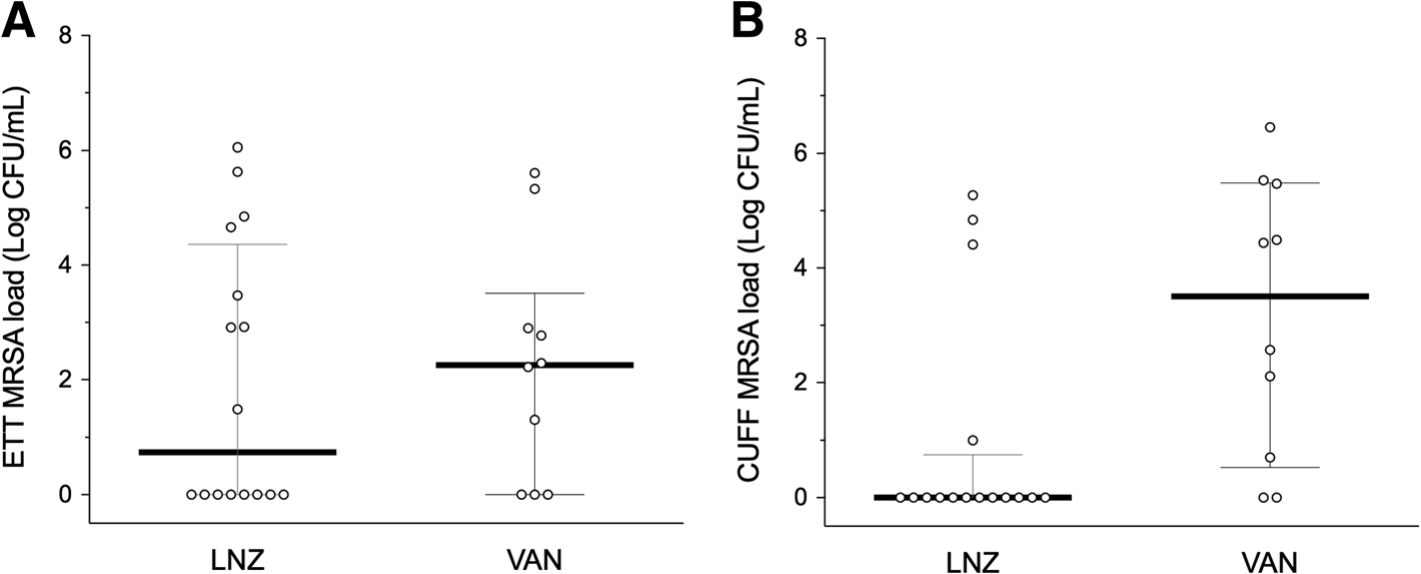
ETT (**a**) and ETT cuff (**b**) MRSA load (log_10_ CFU/mL) in the vancomycin (VAN) and linezolid (LNZ) groups. Each dot represents the MRSA load of each ETT and ETT cuff included into the treatment groups. Black central lines depict median value, while upper and lower gray lines display the 25th and 75th percentiles. Median [IQR] ETT MRSA load was not inferior in the LNZ group (0.74 [0–4.26] vs 2.25 [0–3.21] log_10_ CFU/mL, *p* = 0.83), whereas ETT cuff load was significantly lower in the LNZ-treated patients compared with the VAN treatment group (0 [0–0.75] vs. 3.50 [0.52–5.48] log_10_ CFU/mL, *p* = 0.008). ETT, endotracheal tube; MRSA, methicillin-resistant *S. aureus*; CFU, colony-forming units

We collected all 26 ETT from the patients included. For one reintubated patient, we collected the second tube. When this patient was reintubated, the BAS MRSA count was 5 log_10_ CFU/mL, within the interquartile range of the overall MRSA count at microbial diagnosis in the LNZ arm. There were fewer MRSA-positive ETT cuff cultures in the LNZ than in the VAN group: 4/16 (25%) vs 8/10 (80%, *p* = 0.031) respectively. Similar results were found within ETT, with positive MRSA samples in 8/16 (50%) vs 7/10 (70%), *p* = 0.511 in the LNZ and VAN groups respectively, though the differences were not statistically significant. The MRSA load (log_10_ CFU/mL) of the LNZ group in the ETT cuff was also significantly lower than in the VAN group. However, the MRSA load within the ETT was not significantly lower in the LNZ group (Fig. 2).

No differences were found between LNZ and VAN groups in terms of the presence and load of Gram-positive agents other than *S. aureus* or in Gram-negative bacteria including Enterobacteriaceae, either in ETT or in ETT cuff. The LNZ group showed a higher presence and load of fungi in ETT cuff than the VAN group, although the difference was not statistically significant: 6 (38%) vs 0 (0%) *p* = 0.053 and 1.15 ± 1.71 vs 0.00 ± 0.00 log_10_ CFU/mL, *p* = 0.028, respectively (Additional file 1: Figure S1). LNZ or VAN treatment during intubation did not differ between groups (Additional file 1: Figure S2).

### Emergence of resistance with LNZ and VAN

All *S. aureus* were susceptible to LNZ or VAN in the microbial diagnosis. Susceptibility to LNZ and VAN remained stable in the *S. aureus* strains recruited from the ETT and the ETT cuff.

### LNZ and VAN concentrations in biological matrixes

**Fig. 3:**
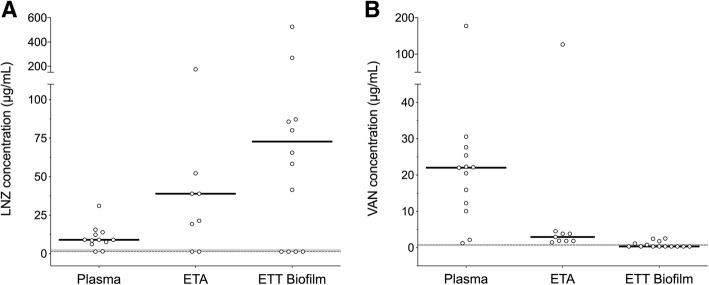
Linezolid (LNZ) (**a**) and vancomycin (VAN) (**b**) concentration in plasma, ETA, and ETT biofilm. In plasma at ETA, LNZ and VAN levels are at 72 h after treatment initiation, in ETT biofilm at extubation. Each dot represents drug concentration of each sample included into the treatment groups. Black central lines depict median value. LNZ median MIC (IQR) was 1.50 [0.88–3.00] μg/mL, while VAN median MIC was 0.75 [0.5–1] μg/mL. The median LNZ and VAN MIC values of the MRSA isolates are indicated by the horizontal gray dashed line while the gray bands represent the 25th–75th percentile ranges in each graph. Of note, LNZ presented high concentrations in ETA and ETT biofilm while VAN concentration fell drastically in ETA and ETT biofilm, to non-therapeutic concentrations. **a** Plasma vs ETA, *p* = 0.039; plasma vs ETT biofilm, *p* = 0.050. **b** Plasma vs ETA, *p* = 0.021; plasma vs ETT biofilm, *p* < 0.001. ETT, endotracheal tube; ETA, endotracheal aspirates; MRSA, methicillin-resistant *Staphylococcus aureus*; MIC, minimum inhibitory concentration

At 72 h after treatment initiation, plasma concentrations of LNZ and VAN were 9.00 [6.51–13.46] vs 22.04 [11.18–26.54] μg/mL, *p* = 0.024 respectively, both figures being above the recommended therapeutic levels (3 μg/mL for LNZ and 15–20 μg/mL for VAN). However, in ETA LNZ was highly concentrated, reaching 38.90 μg/mL [10.22–81.70], while VAN was barely found, at only 2.96 μg/mL [1.86–4.21] (*p* = 0.145) (Fig. 3).

In the LNZ group, seven samples were excluded, three due to absence of biofilm and four due to technical interferences. In the remaining 14 ETT biofilm samples, four were BDL. In general, the overall LNZ concentration within ETT reached high values (72.81 [1.25–127.05] μg/mL), far higher than the median MIC of ETT *S. aureus* (1.50 [1.00–3.00] μg/mL). Nevertheless, VAN was hardly ever found within ETT (0.35 [0.35–1.31] μg/mL, *p* < 0.001), or was found at levels very close to the MIC (0.75 [0.50–1.00] μg/mL) (Fig. 3). Specifically, of 14 VAN-treated ETT biofilm samples, nine were BDL. Moreover, within ETT biofilm, LNZ was found 27.64 [1.25–43.69] median folds above each respective *S. aureus* LNZ MIC, while VAN was found only 0.70 [0.47–2.00] folds above the VAN MIC, *p* = 0.013.

### Clinical outcomes

Ventilatory parameters and gasometry were evaluated at 72 h in both groups (Additional file 1: Table S1). No significant differences between groups were found in length of mechanical intubation or ventilation between LNZ and VAN 9.00[7.00–12.50] and 14.00[8.25–18.75], *p* = 0.169, or 17.50[9.00–29.25] and 25.00[16.25–40.00], *p* = 0.170, respectively (Additional file 1: Table S2).

### Biofilm through SEM

**Fig. 4:**
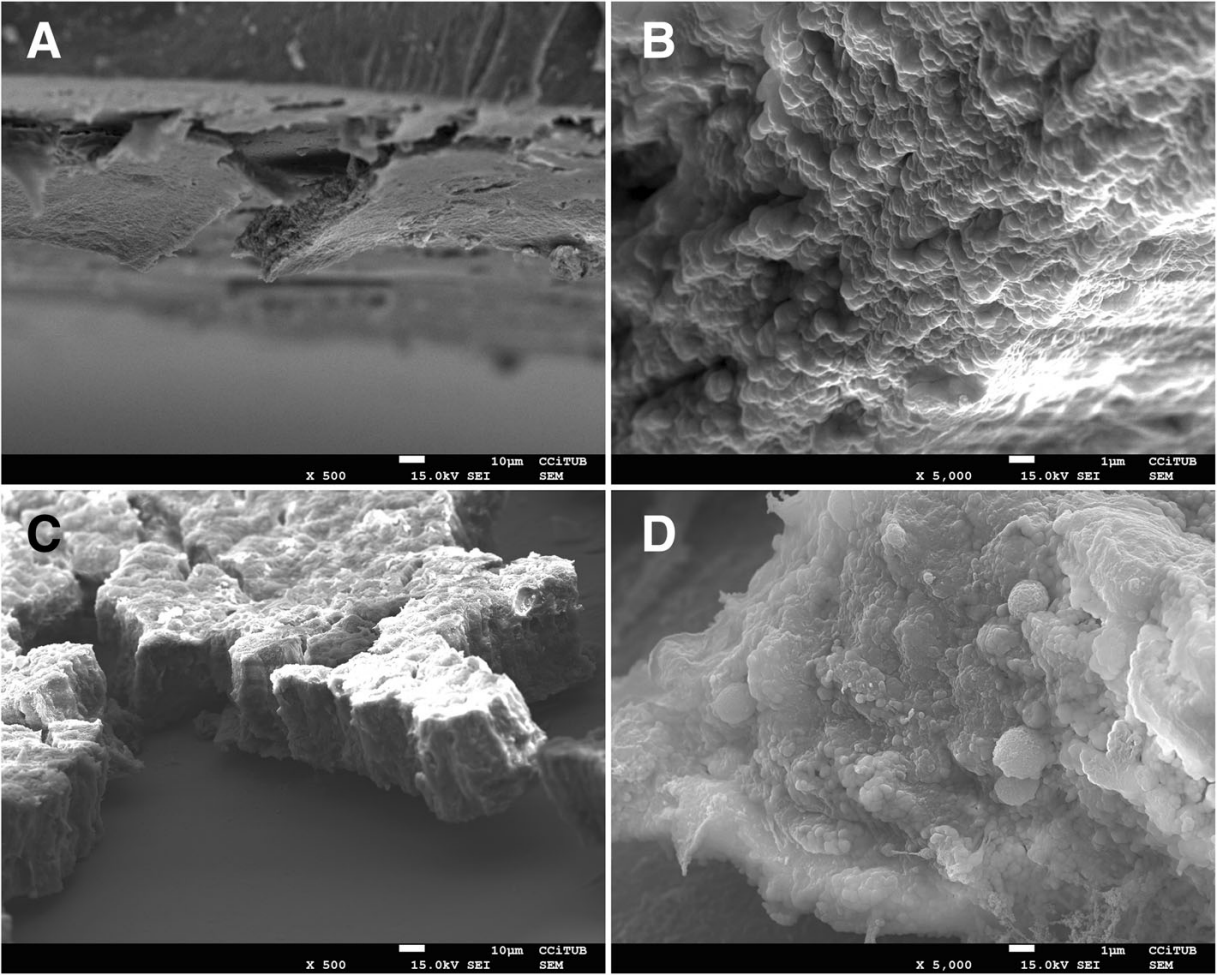
Scanning electron microscopy images of the highest MRSA load ETT biofilm (10^6^ log_10_ CFU/mL) in both groups: vancomycin (VAN) (**a**, **b**) and linezolid (LNZ) (**c**, **d**). Mature biofilms are visible in both groups at low and high magnification. ETT, endotracheal tube; MRSA, methicillin-resistant *Staphylococcus aureus*

Scanning electron microscopy images of ETT biofilm from LNZ and VAN groups are shown in Fig. 4. Overall, within the 15 MRSA-positive ETTs, minimal, maximum, and mean thickness did not differ between LNZ and VAN groups.

### MLST and agr

Different types of ST and agr were identified. The ST22 (33%), ST217 (10%), and ST8 (10%) and agr I (81%), agr III (14%), and agr II (5%) were the most frequent types of MRSA isolated. Each of the other ST (1221, 954, 1535, 83, 403, 3060, 45, 87, 121) was found once (5%).

## Discussion

Systemic treatment of MRSA respiratory infection with LNZ in mechanically ventilated ICU patients resulted in lower ETT biofilm and ETT cuff MRSA burdens than in patients who received VAN. Indeed, MRSA eradication was superior in ETT (50 vs 30%) and ETT cuff (75 vs 20%) in the LNZ than in the VAN group, although the difference was statistically significant only with regard to ETT cuff. Accordingly, the concentration of LNZ was higher than VAN in ETA and also in ETT biofilm, even though both drugs achieved therapeutic plasma levels at 72 h after treatment initiation.

This is the first report in the literature comparing the effects of LNZ and VAN in ETT from mechanically ventilated humans. Our findings indicate that LNZ is more effective in ETT cuff than VAN, since MRSA presence and loads were significantly lower in the LNZ group. Why is this important?

On the one hand, ETT cuff microfolds, formed in contact with the tracheal wall, are considered a common route of microbial access to the lower respiratory airways [27]. For this reason, attempts have been made in order to minimize ETT cuff aspiration of subglottic secretions. Although these systems lose efficacy over time [28–32], their efficacy can be complemented by systemic antibiotics with ETT biofilm and cuff effect like LNZ, but not VAN.

On the other hand, we demonstrated the superiority of LNZ over VAN in ETA and ETT biofilm drug concentration. The efficacy of LNZ penetrating into respiratory secretions is emphasized by the therapeutic levels achieved by both drugs in plasma compared with their concentrations in ETA 72 h after the first drug administration, in which LNZ remained several folds above the MIC but VAN levels remained subtherapeutic in most of the samples. Notably, the concentrations of LNZ and VAN in ETA (72 h) are indicative of their concentration in ETT biofilm after extubation. Nevertheless, the usefulness of ETA (72 h) for predicting other drug concentrations in ETT biofilm after extubation needs to be investigated further.

Although biofilms exhibit intrinsic tolerance to antibiotics [10, 33], in the ETT, the presence of antibiotics and the development of the biofilm are concomitant. This increases the ability of systemic LNZ treatment to limit biofilm development, as its ETT MRSA eradication rate is 67% higher than that of VAN. However, in critically ill patients, the distribution of LNZ within the ETT, mainly driven by respiratory secretions, is not homogeneous; therefore, its efficacy for eradicating MRSA is not always guaranteed.

All the ETT MRSA were susceptible to LNZ and VAN MIC after long periods of intubation. This clearly highlights that the emergence of resistant strains associated with biofilms is less likely in intubated patients, a finding that is at odds with previous findings in other respiratory diseases [34]. All VAN MIC but two were below 1.5 μg/mL, a threshold MIC that has been previously associated with lower clinical response, higher relapse [35], and increased mortality.

In contrast, we did not find differences in biofilm thickness between LNZ and VAN groups. This may be due to differences between secretion production and microbiota in pigs and in human patients. Thus, the proposal that thickness might be a good indicator of treatment efficacy in a highly controlled experiment [12, 13] may not apply to human patients, where the underlying conditions and other concomitant issues may influence the biofilm and secretions accumulated within the ETT, in addition to length of stay and treatment efficacy.

The results of our study corroborate those of many previous randomized clinical trials. The Zephyr study [36] observed higher rates of clinical cure in nosocomial *S. aureus* pneumonia (both MRSA and MSSA) when comparing LNZ to VAN. Surprisingly, the IDSA/ATS guidelines still place VAN and LNZ at the same level [4], even though there are enough clinical and animal data to change this recommendation in favor of LNZ [5]. Our study is also in line with a previous study published by our group in a pig model of MRSA pneumonia in animals ventilated for 72 h. However, findings in animals require replication in humans.

The strengths of our study are the following: (1) this is the first comparison of linezolid and vancomycin in ETT biofilms obtained from humans on long-term mechanical ventilation and (2) ETT (including cuff) biofilms and bacterial burden are studied in depth.

A few potential limitations of this study deserve further clarification. This was not a randomized study, and so there is no possibility of comparing the outcomes. In addition, the fact that we had to recruit patients from different hospitals increased the heterogeneity of the ST types involved. Nevertheless, we did not find any differences in patients’ characteristics or in the length of orotracheal intubation between VAN and LNZ groups, and so the heterogeneity of the MRSA ST collected emphasizes the validity of our results and provides realistic epidemiologic data. Secondly, the use of VAN is becoming less and less frequent in Europe, and for this reason, the number of ETT within this group of study was lower than in the LNZ group. Ultimately, these patients received concomitant antimicrobials that may have combined effects with vancomycin or linezolid. However, concomitant antimicrobials were homogeneously distributed between the two treatment groups.

The main clinical implication of our results is that LNZ, which acts effectively in ETT biofilms and cuffs, performs much better than VAN in MRSA eradication and may be important in preventing relapses in MRSA VAP pneumonia.

## Conclusions

In conclusion, systemic treatment with linezolid exerts a greater beneficial effect than vancomycin, reducing the MRSA burden within ETT cuff in ICU patients with MRSA respiratory infection who are intubated for long periods. This additional benefit of linezolid should be taken into account when choosing the antibiotics to treat MRSA VAP.

## Additional files

Additional file 1: (DOC 401 kb)

## Data Availability

All data generated or analyzed during this study are included in this published article.
