# Supplementary material for: Comparative efficacy of linezolid and vancomycin for endotracheal tube MRSA biofilms from ICU patients
Source: Crit Care. 2019 Jul 10;23:251. doi: 10.1186/s13054-019-2523-5 (PMC6617612; doi:10.1186/s13054-019-2523-5)
Supplement: Supplementary file 1 — (DOC 401 kb) [file 13054_2019_2523_MOESM1_ESM.doc]

Supplementary information

**Comparative efficacy of linezolid and vancomycin for endotracheal tube MRSA biofilms from ICU patients**

Laia Fernández-Barat* (1,2), Ana Motos* (1,2), Mauro Panigada (3), Francisco Álvarez-Lerma (4), Lucía Viña (5), Ruben Lopez-Aladid (1,2), Adrian Ceccato (1,2), Gianluigi Li Bassi (1,2), David P. Nicolau (6), Yuli Lopez (7), Laura Muñoz (7), Laura Guerrero (1), Dolors Soy (1,9), Trinidad Israel (2), Pedro Castro (8), Antoni Torres (1,2)

**AFFILIATIONS**

(1) Cellex laboratory, CibeRes ((Centro de Investigación Biomédica en Red de Enfermedades Respiratorias, 06/06/0028)- Institut d’Investigacions Biomèdiques August Pi i Sunyer (IDIBAPS), School of Medicine, University of Barcelona, Spain

(2) Respiratory Intensive Care Unit Pneumology Department, Hospital Clínic, Barcelona, Spain;

(3) Department of Anesthesiology, Intensive Care and Emergency, Fondazione IRCCS Ca' Granda, Ospedale Maggiore Policlinico, Milan, Italy.

(4) Critical Care Dept, Hospital del Mar, Critical Illness Research Group (GREPAC), Hospital del Mar Medical Research Institute (IMIM), Barcelona, Spain;

(5) Servicio de Medicina Intensiva, Hospital Universitario Central de Asturias, Oviedo, Spain;

(6) Center for Anti-Infective Research and Development, Hartford Hospital, Hartford, CT USA.

(7) Microbiology Department, Hospital Clínic, CRESIB ISglobal, Barcelona, Spain;

(8) Medical Intensive Care Unit, Hospital Clínic, Barcelona, Spain;

(9) Pharmacy Service. Division of Medicines. Hospital Clínic, Barcelona, Spain

* Both authors contributed equally to this work.

RESULTS

**Quantitative microbiology assessment of ETT and ETT-cuff**

No differences were found in terms of presence and load of other Gram positive but *S. aureus*, Gram negative bacteria including Enterobacteriaceae between LNZ and VAN groups, neither in ETT nor in ETT-cuff. LNZ ETT-cuff showed higher presence and load of fungi than VAN group, although slightly statistical significance: 6 (38%) vs 0 (0%) p=0.053 and 1.15±1.71 vs 0.00±0.00 log_10_ CFU/mL, p=0.028, respectively (Figure 1S). LNZ or VAN treatment during intubation did not differed between groups (Figure 2S).

**Clinical Outcomes**

Ventilatory parameters and gasometry were evaluated at 72h in both groups. No differences were found despite lactate levels at 72 were higher in LNZ group (Table1S). No significant differences between groups were found in length of mechanical intubation or ventilation between LNZ and VAN 9.00[7.00-12.50] and 14.00[8.25-18.75], p=0.169 or 17.50[9.00-29.25] and 25.00[16.25-40.00], p=0.170, respectively (Table 2S). Higher number of tracheotomies was observed within the population treated with VAN.

**Figure Legend (Supplementary information)**

**Figure 1S**. Load distribution (log_10_ CFU/mL) of all the species isolated in each ETT (A) and ETT-cuff (B) included in the vancomycin (VAN) and linezolid (LNZ) treatment groups. Bar charts display mean load of all the species isolated from ETT and ETT cuff. No differences were found in terms of presence and load of other Gram positive but *S. aureus* (*Staphylococcus spp, Enterococcus spp, and Streptococcus spp*), Gram negative bacteria including Enterobacteriaceae (*Acinetobacter baumannii, Pseudomonas aeruginosa, Stenotrophomonas maltophilia, Escherichia coli, and Klebsiella pneumoniae*) between LNZ and VAN groups, neither in ETT nor in ETT-cuff. LNZ ETT-cuff showed higher presence and load of fungi than VAN group, although slightly statistical significance: 6 (38%) vs 0 (0%) p=0.053 and 1.15±1.71 vs 0.00±0.00 log_10_ CFU/mL, p=0.028, respectively. ETT, endotracheal tube; MRSA, methicillin-resistant *S. aureus;* CFU, colony-forming units

**Figure 2S.** Treatment exposure during intubation. The duration (days) of LNZ or VAN treatment since treatment initiation upon extubation (A) or during intubation (B), did not present statistical differences comparing LNZ vs VAN groups: 7.50[5.25-11.75] vs 6.50[4.50-10.75] p=0.615, 7.00[3.50-10.50] vs 6.00[4.50-8.50] p=0.577, respectively.

Figure 1S.


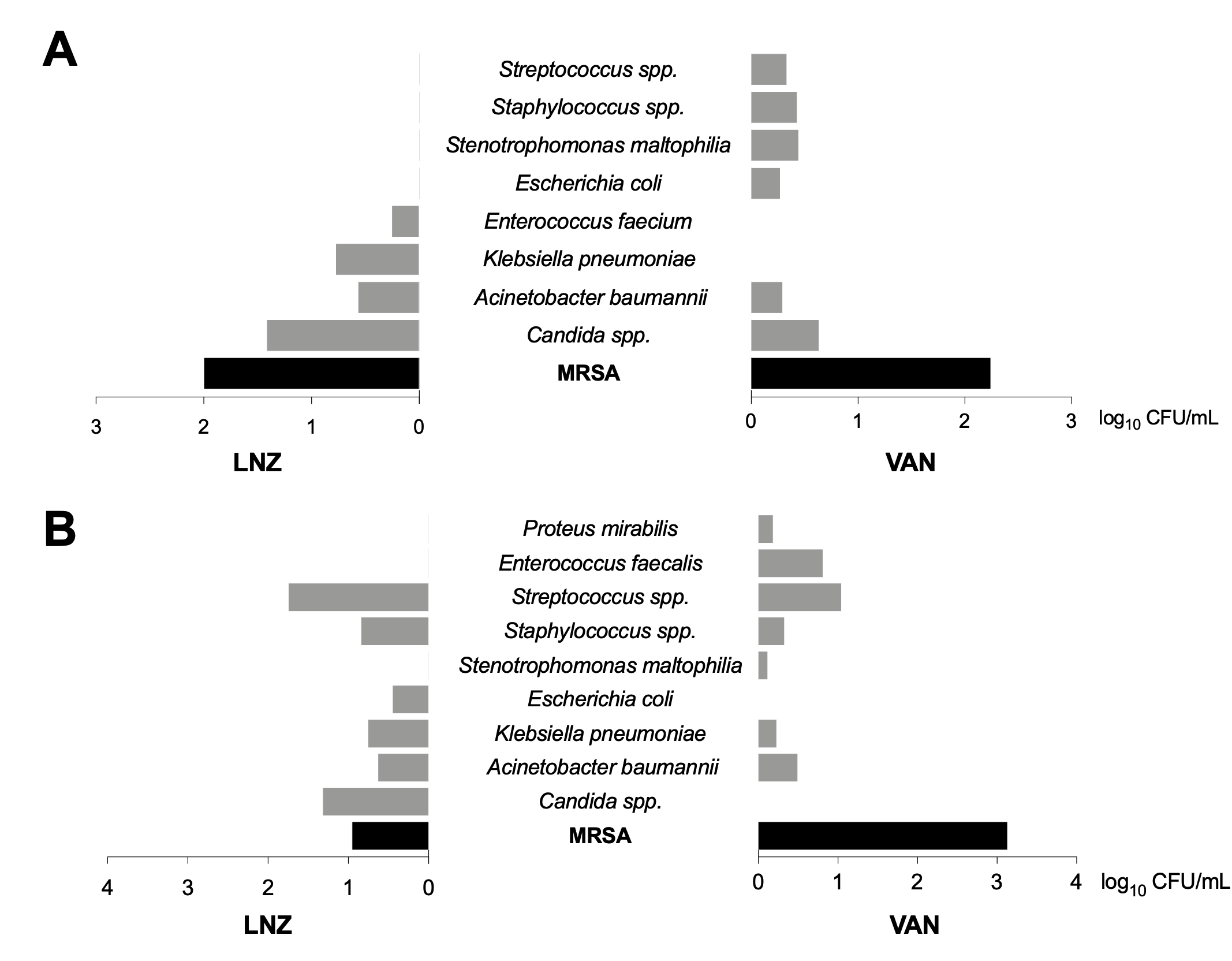


Figure 2S.


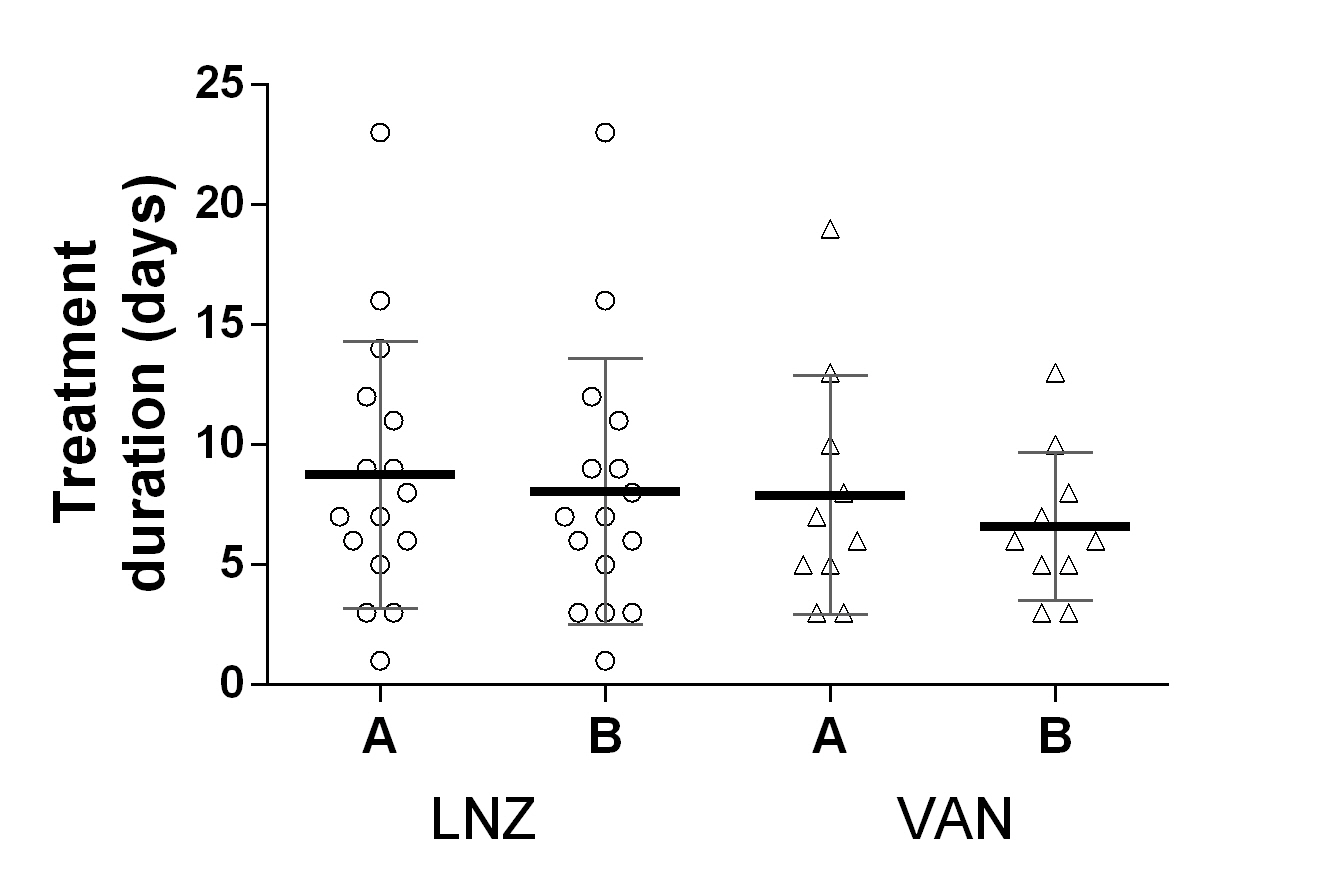


**Table 1S. Follow up at 72 h after antibiotic treatment initiation**

| **MRSA Respiratory ICU infection (n=26)** | **Linezolid (16)** | **Vancomycin (10)** | **p-value** |
| --- | --- | --- | --- |
| **Ventilator settings** |  |  |  |
| V_T_ (mL) | 450[450-512] | 455[415-555] | 0.90 |
| RR (breath/min) | 19.0[18.0-23.0] | 21.0[14.5-24.3] | 0.71 |
| FiO_2_ (%) | 40.0[35.0-60.0] | 40.0[39.0-50.0] | 0.77 |
| PEEP (cm H_2_O) | 10.0[6.0-12.0] | 9.0[7.5-14.3] | 0.73 |
| **Arterial gases** |  |  |  |
| PaO2 (mm Hg) | 87.0[78.0-100.0] | 94.8[70.5-117.5] | 0.51 |
| PaO2/FiO2 (mm Hg) | 180.8[119.6-228.3] | 237.0[131.0-298.0] | 0.10 |
| PaCO2 (mm Hg) | 46.2[33.3-49.8] | 44.5[39.0-49.6] | 0.94 |
| pH | 7.4[7.4-7.4] | 7.4[7.4-7.5] | 0.32 |
| BE (mmol/L) | 1.7[-6.7-5.9] | 2.9[1.5-5.9] | 0.34 |
| Bicarbonate (mmol/L) | 25.0[20.5-31.2] | 27.6[24.4-31.1] | 0.23 |
| Lactate (mmol/L) | 2.1[1.5-2.5] | 1.2[0.7-1.6] | **0.02** |

Data are presented as median and interquartile range [percentiles 25^th^ -75^th^] or n (%).ICU, Intensive care unit; V_T_, tidal volumen; RR, respiratory rate; FiO_2_, inspiratory fraction of O_2_; PEEP, positive end-expiratory pressure; PaO_2_, partial arterial O_2_ pressure; PaCO_2_, partial arterial CO_2_ pressure; BE, base excess;

**Table 2S. Length of mechanical ventilation and reason of extubation**

| **MRSA Respiratory ICU infection (n=26)** | **Linezolid (16)** | **Vancomycin (10)** | **p-value** |
| --- | --- | --- | --- |
| Lenght of orotracheal Intubation (days) | 9.0[7.0-12.5] | 14.0[8.3-18.8] | 0.17 |
| Length of MV (days) | 17.5[9.0-29.3] | 25.0[16.3-40.0] | 0.17 |
| **Reason of extubation (%)** |  |  | 0.33 |
| change of cannula | 1 (6.3) | 0 |  |
| extubation | 5 (31.3) | 1 (10.0) |  |
| reintubation | 1 (6.3) | 1 (10.0) |  |
| tracheostomy | 7 (43.8) | 8 (80.0) |  |
| exitus | 2 (12.5) | 0 |  |

Data are presented as median and interquartile range [percentiles 25^th^ -75^th^] or n (%), ICU: Intensive care unit, MV mechanical ventilation.
